# RNA Polymerase Inhibitor Enisamium for Treatment of Moderate COVID-19 Patients: A Randomized, Placebo-Controlled, Multicenter, Double-Blind Phase 3 Clinical Trial

**DOI:** 10.3390/arm92030021

**Published:** 2024-05-06

**Authors:** Olga Holubovska, Pavlo Babich, Alla Mironenko, Jens Milde, Yuriy Lebed, Holger Stammer, Lutz Mueller, Aartjan J. W. te Velthuis, Victor Margitich, Andrew Goy

**Affiliations:** 1 Department of Infectious Diseases, O.O. Bogomolets National Medical University, T. Shevchenko Blvd. 13, 01601 Kyiv, Ukraine; ogolubovska@gmail.com; 2 State Expert Center, Smolenska Str. 10, 03057 Kyiv, Ukraine; babich@gmail.com; 3 Department of Respiratory and Other Viral Infections, L.V. Gromashevsky Institute of Epidemiology and Infectious Diseases of the NAMS of Ukraine, Amosova Str. 5a, 03083 Kyiv, Ukraine; miralla@ukr.net; 4 Pharmalog Institut für Klinische Forschung GmbH, Oskar-Messter-Str. 29, 85737 Ismaning, Germany; milde@pharmalog.com (J.M.); stammer@pharmalog.com (H.S.); 5 Pharmaxi LLC, Filatova Str. 10A, 01042 Kyiv, Ukraine; lebed@pharmaxi.com.ua; 6 Regenold GmbH, Zöllinplatz 4, 79410 Badenweiler, Germany; lutz.mueller@regenold.com; 7 Department of Molecular Biology, Princeton University, Princeton, NJ 08544, USA; 8 Division of Virology, Department of Pathology, University of Cambridge Addenbrooke’s Hospital, Cambridge CB2 2QQ, UK; 9 Farmak Joint Stock Company, Kyrylivska Str., 04080 Kyiv, Ukraine

**Keywords:** COVID-19, SARS-CoV-2, clinical trial, RNA polymerase, antiviral, FAV00A, Amizon

## Abstract

**Highlights:**

**What are the main findings?**

- Enisamium, administered in conjunction with standard care, demonstrated clinical efficacy in hospitalized adults with moderate COVID-19 requiring supplemental oxygen.
- In patients with moderate COVID-19 requiring supplemental oxygen, treatment with enisamium led to a significant reduction in the time to improvement and a reduction in symptoms compared to placebo, particularly when administered within 4 days of COVID-19 symptom onset.

**What is the implication of the main finding?**

- Enisamium presents a promising treatment option for individuals with moderate COVID-19, offering faster recovery and shorter hospital stays.
- Early administration of enisamium, within 4 days of symptom onset, may lead to more rapid clinical improvement, underscoring the importance of early intervention in managing COVID-19 cases.

**Abstract:**

Enisamium is an orally available therapeutic that inhibits influenza A virus and SARS-CoV-2 replication. We evaluated the clinical efficacy of enisamium treatment combined with standard care in adult, hospitalized patients with moderate COVID-19 requiring external oxygen. Hospitalized patients with laboratory-confirmed SARS-CoV-2 infection were randomly assigned to receive either enisamium (500 mg per dose, four times a day) or a placebo. The primary outcome was an improvement of at least two points on an eight-point severity rating (SR) scale within 29 days of randomization. We initially set out to study the effect of enisamium on patients with a baseline SR of 4 or 5. However, because the study was started early in the COVID-19 pandemic, and COVID-19 had been insufficiently studied at the start of our study, an interim analysis was performed alongside a conditional power analysis in order to ensure patient safety and assess whether the treatment was likely to be beneficial for one or both groups. Following this analysis, a beneficial effect was observed for patients with an SR of 4 only, i.e., patients with moderate COVID-19 requiring supplementary oxygen. The study was continued for these COVID-19 patients. Overall, a total of 592 patients were enrolled and randomized between May 2020 and March 2021. Patients with a baseline SR of 4 were divided into two groups: 142 (49.8%) were assigned to the enisamium group and 143 (50.2%) to the placebo group. An analysis of the population showed that if patients were treated within 4 days of the onset of COVID-19 symptoms (*n* = 33), the median time to improvement was 8 days for the enisamium group and 13 days for the placebo group (*p* = 0.005). For patients treated within 10 days of the onset of COVID-19 symptoms (*n* = 154), the median time to improvement was 10 days for the enisamium group and 12 days for the placebo group (*p* = 0.002). Our findings suggest that enisamium is safe to use with COVID-19 patients, and that the observed clinical benefit of enisamium is worth reporting and studying in detail.

## 1. Introduction

Severe acute respiratory syndrome coronavirus 2 (SARS-CoV-2) [1] was identified in December 2019 as the cause of a respiratory illness designated coronavirus disease 2019 (COVID-19) [2]. Early in the pandemic, various new and repurposed antiviral drugs were considered as treatments for COVID-19 and evaluated in clinical trials, including remdesivir, favipiravir, azithromycin, hydroxychloroquine, and ritonavir, among others [3,4]. Remdesivir (Veklury), a combination of nirmatrelvir with ritonavir (Paxlovid), and molnupiravir (Lagevrio) have shown clinical efficacy in reducing mortality, the need for mechanical ventilation, and improving the clinical status of COVID-19 patients admitted to a hospital, and they are now commercially available through, e.g., patient assistance programs in the United States. Other approved and considered treatments include Ensitrelvir [5], VV116 [6], and non-steroidal anti-inflammatory drugs (NSAIDs) [7]. However, research in alternative antivirals remains essential as SARS-CoV-2 variants containing single- or even dual-resistance mutations have been observed, such as in long-term patients [8].

SARS-CoV-2 is a betacoronavirus and member of the order of Nidovirales [1]. SARS-CoV-2 has a positive-sense, non-segmented RNA genome of around 30 kilobases. The first two overlapping open reading frames (ORF) of the genome are translated into two large polyproteins by host cell ribosomes. Following translation, the two polyproteins are cleaved through intrinsic proteolytic activity into 16 non-structural proteins (NSP) to form a membrane-associated replication–transcription complex (RTC) [9,10]. The RTC synthesizes full-length negative-sense viral RNAs, subgenomic negative-sense viral RNAs, and subgenomic, and full-length positive-sense viral RNAs containing a 5′ m7G capped and 3′ polyA-tail [11]. The multiple enzymatic functions of SARS-CoV nsps make the RTC an important drug target. Of particular interest is the primer-dependent viral RNA polymerase activity [12], which resides in nsp12 [9,13].

**Figure 1:**
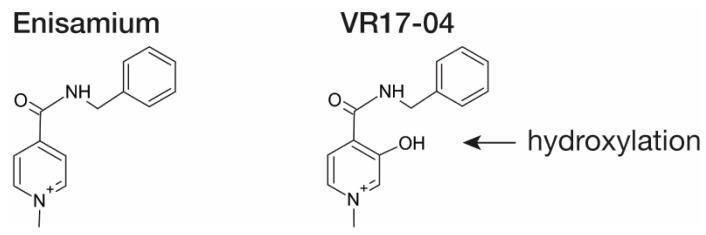
Structure of enisamium and its active metabolite VR17-04.

Early in the COVID-19 pandemic, the World Health Organization (WHO) identified enisamium (4-(benzylcarbamoyl)-1-methylpyridinium (Figure 1), trade name Amizon^®^ MAX), as a candidate drug for the treatment of COVID-19. Enisamium is approved for the treatment of influenza in 11 countries and for the treatment of COVID-19 (in hospitalized patients with moderate disease) in Ukraine [14]. A recent study found that enisamium is efficiently hydroxylated in humans and human lung cells to an active compound called VR17-04 (Figure 1). VR17-04 was previously shown to inhibit the activity of the influenza virus RNA polymerase in vitro [15]. A phase 3 clinical trial provided evidence that enisamium treatment reduces viral shedding and improves patient recovery in influenza patients [15]. Toxicology studies found no genotoxic effects of enisamium in an Ames test, no clastogenic activity in human peripheral lymphocytes with and without metabolic activation, no effect on the incidence of chromosome aberrations at any concentration, and no clinical signs of toxicity or cytotoxicity in bone marrow or micronuclei in Wistar rats at any dose. Following the promising results of enisamium in the treatment of influenza virus infection [14,15,16], we and others recently reported that enisamium can inhibit SARS-CoV-2 replication in Caco-2 and NHBE cells in vitro, while molecular dynamic simulations have suggested that the active compound of enisamium, VR17-04, can bind to the catalytic site of the SARS-CoV-2 RNA polymerase nsp12 [17,18].

The purpose of this study was to evaluate the efficacy and safety of enisamium in hospitalized patients with moderate COVID-19. To this end, we performed a phase 3 clinical trial with adaptive interim analyses [19] at 14 clinical centers in Ukraine. The data contributed to the approval to use enisamium for the treatment of COVID-19 (in hospitalized patients with moderate disease) in Ukraine and several other countries.

## 2. Methods

### 2.1. Study Approval

From 15 May 2020 to 26 March 2021, we conducted a multicenter, double-blind, placebo-controlled, randomized, comparative parallel study at 14 clinical centers across Ukraine. The protocol and materials of the clinical trial were approved by the CEB/Ministry of Health (MOH) of Ukraine under approval number 2949 on 18 December 2020. The study was also approved by the Ethics Committees at the treatment and prevention facilities where the study was conducted. The study was conducted in accordance with the Declaration of Helsinki, the International Principles for Clinical Trials (ICH GCP), the current legislation of Ukraine, and the approved study protocol. All patients provided written informed consent to participate in the study. This trial was registered with ClinicalTrials.gov under NCT04682873.

### 2.2. Study Design

Since COVID-19 was insufficiently studied during our study design and at the start of our study in May 2020, it was difficult to predict the effect of enisamium on COVID-19 patients and choose the right patient group. To have the most impact on COVID-19 patients in Ukraine, we initially set out to study the effect of enisamium on patients with a baseline severity score (SR) of 4 or 5 on an 8-point severity scale (Table 1) and randomized approximately 700 patients to ensure that we could include at least 398 patients in the ITT population (199 for each treatment group). According to the adaptive design principle, we scheduled a conditional power (or interim) analysis in accordance with Bauer–Köhne’s 2-stage approach and stopping rules [20] (please see the section below, “Statistical analysis”).

Based on the interim results, the original protocol was modified and only the recruitment of COVID-19 patients with a baseline of SR = 4 was continued, whereas the recruitment of COVID-19 patients who had a baseline of SR = 5 was stopped. These changes were approved by the CEB and the Ethics Committees at the treatment and prevention facilities where the study was conducted. We will refer to this latter group as the intention-to-treat (ITT) population from here on, and the efficacy analyses presented in this study were for this ITT population only. Data about the SR = 5 patients are censored and not included in the efficacy analysis presented in this publication. Safety data about the SR = 5 patients are included.

### 2.3. Outcomes

The primary endpoint of this study was the time until the improvement of the patient’s condition by 2 points (from 4 to 6) on the 8-point severity scale (Table 1), as measured through an assessment of the patient’s condition. Before data were unblinded, a decision was taken to divide patients into age groups (“<40 years”, “40–<65 years”, and “≥65 years”) similar to the analysis of the effect of remdesivir treatment on COVID-19 patients [21]. A >50-year subgroup analysis was included as a post hoc analysis.

In addition to the primary endpoints, secondary endpoints were analyzed. These secondary endpoints were predetermined. Cough, respiratory rate, and fever were part of the time-to-recovery analysis and recorded based on a verbal rating scale (VRS-4): 0 points—none/not present; 1 point—mild; 2 points—moderate; 3 points—severe. The post hoc analyses presented were based on the same predefined criteria.

### 2.4. Criteria for Inclusion or Exclusion

**Figure 2:**
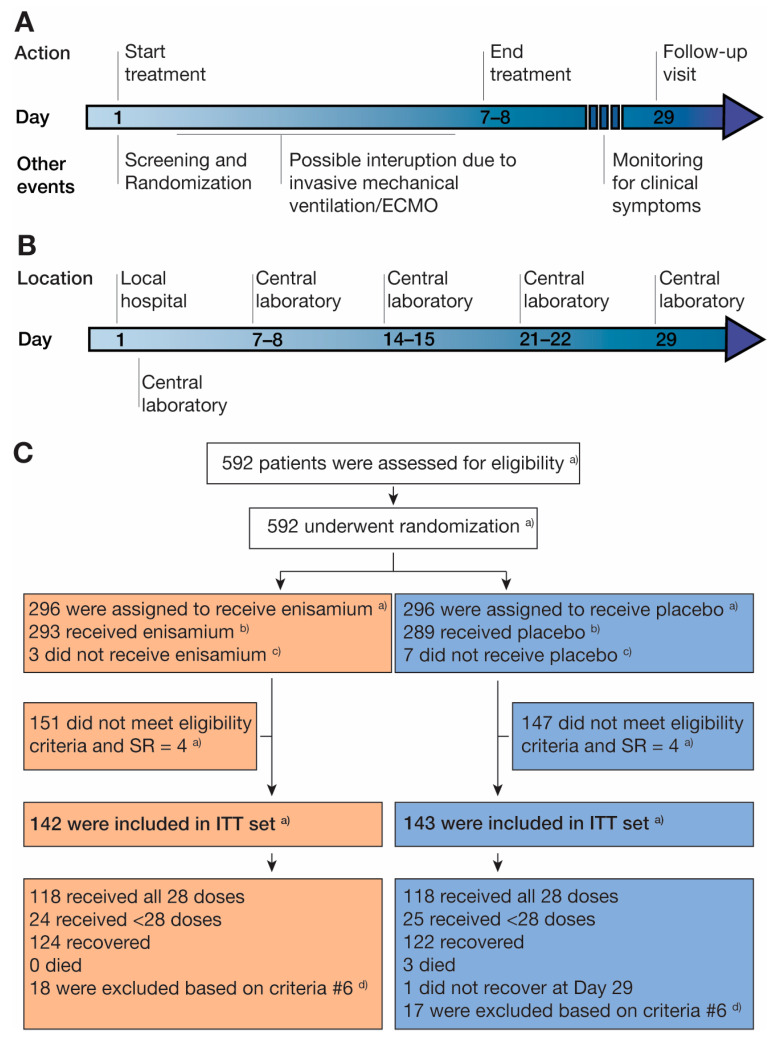
Overview of patient recruitment, randomization, testing, and treatment. (**A**) Timeline of patient recruitment, randomization, and treatment. (**B**) Timeline of patient testing. (**C**) Schematic overview of patient recruitment, randomization, and treatment. (a) This number of patients was included according to both versions of the study protocol. The first version of the protocol allowed the inclusion of patients with an SR = 4 and SR = 5. Patients with an SR = 4 were included in the active and control groups. Patients with an SR = 5 were not included in the efficacy analysis, but they were included in the safety analysis. (b) Patients who received enisamium or placebo were included in the efficacy and safety analyses, while (c) those who did not were excluded. (d) Presence of renal dysfunction. These data were obtained from a patient’s medical history, which was, for some patients, disclosed to the researchers after visit 1 and randomization. In these situations (with evidence of renal dysfunction prior to enrolment), the enrolled patient was removed from the ITT population.

For inclusion in the study, each patient had to meet the inclusion criteria listed in Table S1. Patients were excluded from participation in the study if they met any of the criteria listed in Table S2. A total of 592 patients were assessed based on the 8-point severity scale (Table 1). This total number of patients encompassed both versions of the study protocol. Briefly, the first version of the protocol allowed for the assessment of patients with an SR = 4 or an SR = 5. The second version of the protocol only allowed the assessment of patients with an SR = 4. Moreover, in accordance with the second version of the study protocol, patients with an SR = 5 could not be included in the efficacy analysis and could only be analyzed for the drug safety assessment. All patients who received treatment were included in the safety analysis (see Figure 2C). Note that under exclusion criterion number 6 (Table S2), the data from several patients were censored when their medical history became available after randomization (see below for information on randomization). This exclusion criterion concerns the presence of renal dysfunction, which we defined as follows: eGFR < 60 mL/min, total bilirubin ≥2.0 mg/dL, TSH outside the normal range, and/or ASAT/ALAT above a three-fold upper limit of the normal range. These data were obtained from a patient’s medical history, which, for some patients, was disclosed to the researchers after visit 1 and randomization. In these situations (with evidence of renal dysfunction prior to enrolment), the enrolled patient was removed from the ITT population. For safety reasons, these patients discontinued treatment, but they were counted as ITT because they were recorded as randomized for treatment.

### 2.5. Randomization

At the screening stage, SARS-CoV-2 infection was confirmed via RT-qPCR in hospitalized patients with a diagnosis of COVID-19. Patients who met the criteria for inclusion and did not meet the criteria for exclusion (Table S2) were randomized 1:1 into a group receiving an oral administration of enisamium and a group receiving an oral administration of placebo. 

### 2.6. Procedures

On day 1, patients were screened, randomized, and started on the treatment (Figure 2A,B). COVID-19 nucleic acid tests (see below) were used by the investigator to determine patient inclusion in the study, evaluate a patient’s clinical status, and decide the endpoint of hospitalization. The COVID-19 tests for recruitment were performed by the local hospital to proceed to the randomization and treatment as quickly as possible. The COVID-19 testing of baseline samples was performed by the central testing lab L.V. Gromashevsky Institute of Epidemiology and Infectious Diseases of the NAMS of Ukraine. On days 2 to 7 (or days 2 to 8, if the 168 h of treatment ended on day 8 depending on the time of day that the treatment was started), patients were treated, monitored, and evaluated, provided that the patient had not been discharged from the hospital. Treatment with enisamium or placebo could be stopped if a patient was transferred to mechanical ventilation. All patients received any other treatment deemed necessary by the investigator, depending on the patient health status (“standard of care”). These treatments included glucocorticosteroids (Table S3), although the use of these drugs was not required in Ukraine at the time of our study.

The first dose of enisamium or placebo was administered after the randomization procedure on visit 1 (day 1) and as soon as possible after the onset of symptoms (Table S4). Patients subsequently received enisamium or placebo 4 times a day (4 × 1 capsules) every 6 h. The total treatment duration was 168 h (7 days). On days 8 to 29, no treatment with enisamium or placebo was administered, but patients continued to be observed and evaluated for clinical symptoms if they had not yet been discharged from the hospital. For patients discharged before day 29, a follow-up visit was conducted on day 29 for a final evaluation (Figure 2A,B). Changes in clinical status were summarized on a continuous scale using means, standard deviations (SD), medians, interquartile ranges (IQR), and ranges. Sample analysis from later time points were performed by the central testing lab (Figure 2B). The clinical sites could test patients for the presence of SARS-CoV-2 virus at their discretion throughout their stay at the hospital.

All safety laboratory analyses (hematology, coagulation, clinical chemistry, urinalysis) were performed by the central laboratory, L.V. Gromashevsky Institute of Epidemiology and Infectious Diseases of the NAMS of Ukraine. Additional laboratory evaluations at the local laboratory of each site were performed as deemed necessary by the investigator and in accordance with the local standard of care. The results of these tests were not provided to the sites but used for the efficacy assessment of the clinical study.

### 2.7. Molecular Testing

Throat swabs or sputum tests were taken for SARS-CoV-2 RNA detection using COVID-19 TaqPath kits (Catalog number A48067, Thermo Fisher) on day 1, and if the patient remained hospitalized, on day 8 (±1 day), day 15 (±1 day), day 22 (±1 day), and day 29 (±1 day), or on the day of discharge from the hospital, if discharge occurred earlier than the planned analysis points. Negative and positive controls were included in all molecular tests. During the study, routine throat swabs and sputum collections were performed for SARS-CoV-2 RNA detection and in accordance with the standard COVID-19 management protocols approved by the MoH of Ukraine. These samples were analyzed in the central laboratory L.V. Gromashevsky Institute of Epidemiology and Infectious Diseases of the NAMS of Ukraine (Figure 2A). The COVID-19 test results were used to determine patient inclusion in the study, evaluate a patient’s clinical status during hospitalization, and decide the endpoint of hospitalization. They were considered an important readout [22].

### 2.8. Statistical Analyses

Bauer–Köhne’s 2-stage approach and stopping rules for an adaptive design were used [20]. This method is based on the observed error probabilities from the disjoint subsamples before and after the interim analysis. Formally, an intersection of individual null hypotheses is tested by combining the two *p*-values into a global test statistic. Stopping rules for Fisher’s product criterion in terms of critical limits for the *p*-value in the first subsample are introduced, including early stopping in the case of an absence of promising treatment effects. If no stopping rules apply, the sample size should be recalculated based on the interim results. For our study, we wanted to assess whether the originally planned study was sufficiently promising to reach the primary endpoint in either the SR = 4 or 5 group, or both, and whether more patients could be recruited to either group or whether the study should be terminated for one or both groups.

When over 50% of the patients for the initial protocol design had been recruited (77 patients with a baseline of SR = 4 and 298 patients with a baseline of SR = 5), the preplanned conditional power analysis was performed. The interim analysis showed no promising result for the whole analysis population in the primary endpoint. Further subgroup investigations of the interim data revealed that no relevant treatment difference could be observed in patients with a baseline of SR = 5. However, patients with a baseline of SR = 4, i.e., patients suffering from a higher degree of COVID-19 at baseline, showed a promising benefit from the enisamium treatment compared to the placebo at the primary endpoint.

In order to confirm that the observed trend would continue for the ITT group (and guarantee continued protection for the COVID-19 patients), a second interim analysis was planned after approximately 50% of the preplanned ITT population (i.e., 200 subjects with a baseline of SR = 4) had completed the study. Following data clearance and the adjustment of the statistical model based on the results of the conditional power analysis, a one-sided *p*-value lower than the Bauer–Köhne cut-off of *p* = 0.0131 in the primary endpoint was needed to show a significant benefit of enisamium treatment. Since we found *p* < 0.0131 between the enisamium and placebo populations in the second analysis, the clinical trial was terminated, and the data for the ITT group were unblinded.

Our interim analysis method was based on the observed error probabilities from the disjoint subsamples before and after the interim analysis. In accordance with the applied adaptive research design, an inflation of the level of significance was taken into account when formulating the conclusions of this study [23]. A conditional power analysis was conducted by an independent data monitoring committee, and all procedures were performed in accordance with ICH Guideline E9, “Statistical Principles for Clinical Trials”.

All primary and safety analyses were based on the ITT principle and only performed on the ITT group (baseline of SR = 4). The primary endpoint analysis was performed using Kaplan–Maier curves, hazard function curves, medians of the time to event, and a logrank test. An analysis by patient age was not defined in the protocol when this was submitted early in the COVID-19 pandemic. However, as evidence for a correlation between age and COVID-19 severity accumulated over the course of the pandemic and age group distributions were used in other studies, we considered it prudent to consider age in our analysis. Before data unblinding, a decision was taken to divide patients into age groups (“<40 years”, “40–<65 years”, and “≥65 years”) similar to the analysis of the effect of remdesivir treatment on COVID-19 patients [21]. Indeed, at the blind review stage, differences in the time to clinical improvement were observed between the age categories, regardless of treatment (Tables S5–S7, Figure S1).

For the analysis of secondary endpoint descriptive statistics, graphics methods, an Fisher exact test, a logrank test, Cox regression, and confidence intervals were used. For assessing the superiority of enisamium relative to placebo, we performed one-sided statistical hypothesis testing and set a significance level of 0.0131 for primary endpoints (see above). A significance level of 0.025 was used to perform the one-sided statistical hypotheses for secondary endpoints. Student’s *t*-tests were used to compare independent samples, while the Mann–Whitney test or Fisher’s exact test was used to test the initial homogeneity of the patient group, depending on the nature of the data and distribution. In the analysis of the initial homogeneity of the groups, a significance level of 0.05 (bilateral) was used. All statistical calculations were performed according to the principles of the applied adaptive research design, and the inflation of the level of significance was taken into account during the analysis. The >50-year age group was analyzed using a cluster analysis.

### 2.9. Role of Funding Source

The funders of the study had no role in data collection, data analysis, and data interpretation. VM and AG of Farmak JSC were involved in the study design and writing of the manuscript.

## 3. Results

### 3.1. Patients

A total of 592 patients were tested for the presence of SARS-CoV-2 RNA and randomized, of whom 296 were assigned to the placebo group and 296 to the enisamium group (Figure 2C). The safety and tolerability analysis (SA) population included all patients who received a dose of enisamium or placebo at least once, with 289 patients in the placebo group and 293 in the enisamium group, and thus a total of 582 patients. Given that after the first interim analysis, we decided to focus on subjects whose baseline score was SR = 4 and those who received at least one dose of enisamium in combination with standard care or placebo in combination with standard care (see Methods), the ITT population included 285 subjects, of which 143 subjects had been assigned to the placebo group, and 142 subjects, to the enisamium group (Figure 2C). Some patients included in the ITT population met exclusion criterion 6 (Table S2; Figure 2C) during the study and were subsequently excluded from further participation for safety reasons (for more information, see the section “Randomization”). These patients were included in the efficacy evaluation for the time they were in the study and received the study drug. The patient distribution and exclusions are illustrated in Figure 2C.

The ITT population was 47.0% male and 53.0% female (Table 2). The median age of the ITT population was 59 years (IQR 47–65), and across the three age groups, 11.2% were <40 years, 61.1% were 40 –<65 years, and 27.7% were ≥65 years (Table 2). All patients were from Ukraine, 99.6%-Caucasian (white). Most patients had either one (36.5%) or two or more (26.7%) of the prespecified coexisting conditions at enrollment. The most common comorbidities were hypertension (49.1%), BMI ≥ 30 kg/m^2^ (33.0%), and type 2 diabetes mellitus (9.1%). The median number of days between symptom onset and randomization was 8 days (IQR 6–12).

### 3.2. Primary Endpoint

The ITT population consisted of 285 COVID-19 patients with a baseline SR of four points on an eight-point severity scale (Table 1). The point of clinical improvement was set at an SR of six points. When estimating the number of days before reaching the moment of clinical improvement, all days were counted, including the first day of the patient’s stay in the study. The day on which the patient’s condition reached an SR of six points was not included. Patients who died (SR = 1) were considered in the analysis as those who did not achieve clinical improvement during the entire observation period (29 days). Similarly, patients who did not achieve clinical improvement (i.e., remained in a stable condition, improved in their condition by only one point, and those that declined in their condition) within 28 days were considered as patients who remained in the study for 29 days. Over the course of this study, the patients in the enisamium group all survived, while in the placebo group, three patients died, and one patient remained at SR = 4 on day 29 of the study (Table 3).

**Figure 3:**
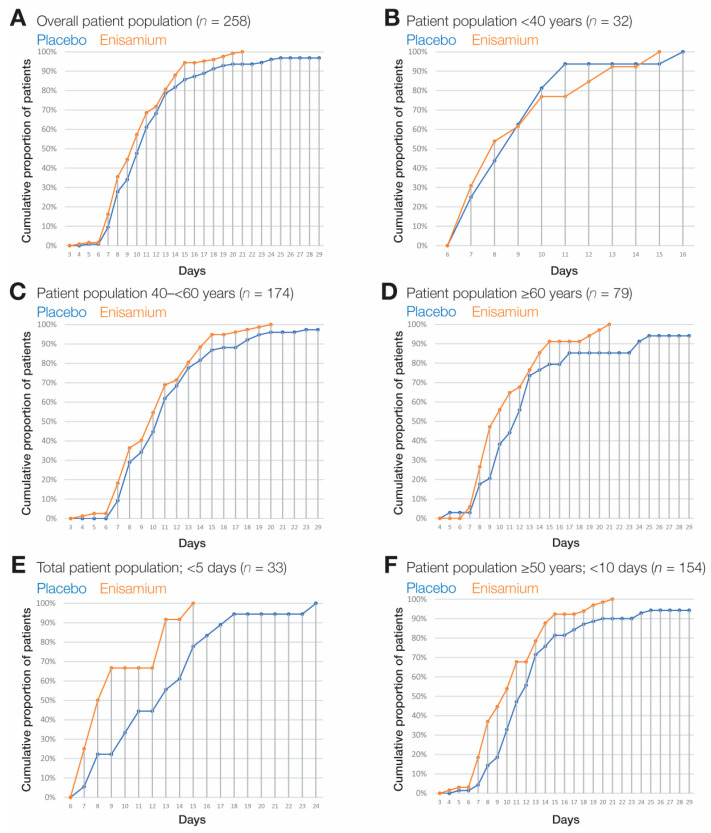
Kaplan–Meier plots. Kaplan–Meier estimates of the cumulative proportion of patients that achieved the primary endpoint in (**A**) the overall patient population (*n* = 258), (**B**) patients in the age category “<40 years” (*n* = 32), (**C**) patients in the age category “40–<65 years” (*n* = 174), (**D**) patients in the age category “≥65 years” (*n* = 79), (**E**) patients randomized within <5 days of symptom onset (*n* = 33), and (**F**) patients aged ≥ 50 years randomized within <10 days of symptom onset (*n* = 154).

Overall, patients in the enisamium group reached the primary endpoint after a median of 10 days compared to a median of 11 days for patients in the placebo group (Figure 3A). Because the differences between the enisamium and placebo group were significant (*p* < 0.0131), this study was stopped in accordance with the protocol. These differences were also present and significant in the stratified age categories (one-sided logrank *p* = 0.00945). Patients <40 years reached the primary endpoint with a median of 8 days in the enisamium group and 9 days in the placebo group. In the 40–<65 years group, the medians were 10 and 11 days, respectively, and in the ≥65 years group, 10 and 12 days, respectively (Figure 3B–D, Table 3).

### 3.3. Subgroup Analyses

Among patients who were randomized <5 days after symptom onset, the enisamium group reached the primary endpoint after a median of 8 days compared to a median of 13 days for patients in the placebo group (*p* = 0.005; 33 patients) (Figure 3E, and Table 3). Among patients aged ≥50 years who had been randomized within <10 days of symptom onset, the enisamium group needed a median of 10 days to reach the primary endpoint, whereas patients in the placebo group required a median of 12 days (one-sided *p* = 0.002; 154 patients) (Figure 3F and Table 3).

### 3.4. Secondary Endpoints

**Figure 4:**
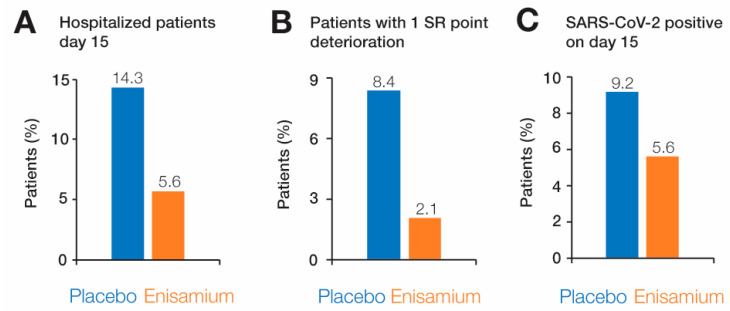
Patients testing positive or showing a deterioration in their SR score. (**A**) Percentage of patients on day 15. (**B**) Percentage of patients with a change in SR from 4 to 3 after randomization. (**C**) Percentage of patients testing positive for SARS-CoV-2 RNA via RT-qPCR on day 15, which is representative of the other days.

During the trial, we recorded secondary endpoints, including “discharge from hospital on day 15”, “discharge from hospital on day 22”, and “prevention of deterioration after randomization”. We observed that on day 15, significantly fewer patients remained in the hospital in the enisamium-treated group compared to the placebo group (5.65% vs. 14.29%; one-sided *p* = 0.018) (Table 4, Figure 4A). On day 22, there were 0% hospitalized patients in the enisamium group compared to 6.3% in the placebo group (0.0% vs. 6.3%; one-sided *p* = 0.004).

We also kept track of the need for oxygen support. The need for stronger oxygen support was assessed by calculating the frequency of and time required for the deterioration of the patient by one point on the eight-point severity scale (i.e., from SR = 4 to SR = 3), as patients with the deteriorated condition had to receive non-invasive or high-flow oxygen therapy. The proportion of patients who deteriorated was 8.4% in the placebo group and 2.1% in the enisamium group, which is significantly better in the enisamium group compared to the placebo group (*p* = 0.016; one-sided) (Table 4, Figure 4B). If we evaluate the ratio of the chances of preventing the deterioration of the patient’s condition in the enisamium group compared with the placebo group, we obtain an OR = 4.244 (95% CI: 1.171–15.380). Thus, we conclude that the use of enisamium increases the chances of preventing the deterioration of patients by about four times compared with placebo.

Finally, we analyzed the levels of SARS-CoV-2 via RT-qPCR, an important virological readout. We measured SARS-CoV-2 RNA levels throughout the trial at days 8, 15, 22, and 19 (Figure 1B). As shown in Figure 4C and Table 4, we observed fewer SARS-CoV-2 RT-qPCR results in the enisamium group compared to the placebo group at all four sample days, but this difference was not statistically significant (Table 4, Figure 4C). All results for secondary endpoints and other secondary endpoints are listed in Table 4, Tables S8 and S9.

### 3.5. Symptom Dynamics

**Figure 5:**
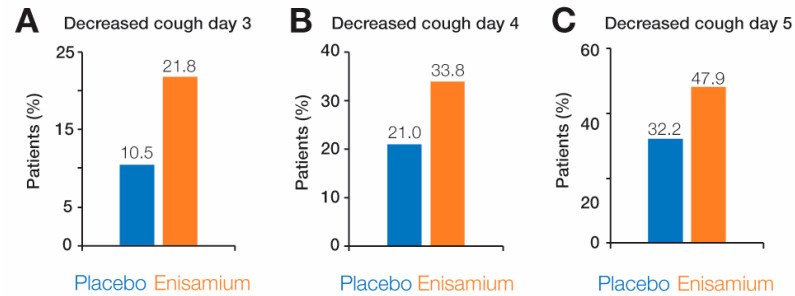
Proportions of patients with decreased cough on days 3 to 5. (**A**) Proportion of patients with decreased cough on day 3, (**B**) day 4, and (**C**) day 5.

At the start of the study, 98.4% of subjects in the placebo group and 97.2% of subjects in the enisamium group had a cough of varying severity (mild, moderate, or severe on the VRS-4 score). On days 3, 4, and 5 after the initiation of treatment, the proportion of patients who demonstrated decreased cough severity was statistically significantly higher in the enisamium group compared to the placebo group (day 3: 21.8% vs. 10.6%, *p* = 0.007; day 4: 33.8% vs. 21.1%, *p* = 0.011; day 5: 47.9% vs. 32.4%, *p* = 0.005). No statistically significant differences were observed on the other study days. In addition, we observed no statistically significant differences for other recorded symptoms, which included rhinorrhea, sore throat, headache, shortness of breath, diarrhea, myalgia, and fatigue (Figure 5, Table S10).

### 3.6. Safety Outcomes

The SA population (289 patients in the placebo group and 293 in the enisamium group) included 582 randomized patients who had each received at least one dose of the study drug or placebo control. A total of 28 doses were taken by 80.6% of the patients in the placebo group and 80.2% of the patients in the enisamium group. This study found 172 adverse reactions/adverse events (ARs/AEs) in 87 placebo subjects and 229 ARs/AEs in 105 patients in the enisamium group (Table 5). Most ARs/AEs were mild and moderate in both the placebo and enisamium groups. The physician classified causation as “related” for 24.4% of ARs/AEs in the placebo group and for 48.9% of ARs/AEs in the enisamium group. The ARs/AEs in the enisamium group that were associated with the study drug were mild or moderate and did not require additional treatment, and therefore, the safety of enisamium can be considered good. The investigators rated the overall tolerability of enisamium as very good (45.3%), good (49.1%), and moderate (5.6%) in the randomized patients. Differences in tolerability estimates between the placebo and enisamium groups were not statistically significant (*p* = 0.289; two-sided). Patients rated the overall tolerability of enisamium as very good (50.6%), good (43.4%), and moderate (5.6%). Differences in tolerability estimates between the two groups were not statistically significant (*p* = 0.260; two-sided).

The study did not reveal negative clinically significant dynamics of the laboratory parameters of the complete blood count (CBC) and other blood parameters (leukocytes, erythrocytes, hemoglobin, hematocrit, lymphocytes, monocytes, neutrophils, eosinophils, basophils, platelets, mean corpuscular hemoglobin (MCH), mean corpuscular volume (MCV) and mean corpuscular hemoglobin concentration (MCHC)) in either group. In addition, this study did not reveal clinically significant dynamics of the laboratory parameters of the general analysis of urine and did not reveal clinically significant changes in the laboratory parameters of biochemical blood tests (ALT, AST, glucose, total bilirubin, creatinine, cholesterol, LDL, GGT, potassium, sodium, calcium, triglycerides, free thyroxine, and free thyroxine) in either group. Changes in these indicators during the study were random, and statistically and clinically insignificant. A normalization of the laboratory parameters was also observed and attributed to improvement in the clinical condition.

## 4. Discussion

Studies performed during the COVID-19 pandemic have shown that RNA polymerase inhibitors are important antivirals when administered soon after symptom onset. Indeed, various treatments, Paxlovid [24], Ensitrelvir [5], and others are now available to COVID-19 patients. However, it remains important to find and characterize additional antivirals, as resistance mutations, and even dual resistance mutations, have been observed in COVID-19 patients, particularly in long-term patients [8], and limitations exist in our ability to predict the clinical efficacy of drugs based on laboratory experiments [25]. We previously investigated the effect of enisamium on influenza patients and found that enisamium treatment improved patient recovery when compared to a placebo control [15]. No other studies have described an assessment of the clinical efficacy of enisamium for the treatment of COVID-19 so far.

Our data indicate that enisamium provides a benefit to the recovery of patients with a baseline score of SR = 4, i.e., hospitalized patients receiving oxygen support, relative to patients receiving placebo (Figure 3). The analysis of the secondary results showed reduced SARS-CoV-2 positivity in the enisamium-treated group at all time points (Table 4), an important measure in virological studies. However, the difference observed was not significant. It is possible that the lack of significance derives from non-infectious SARS-CoV-2 RNA, which can remain present long after patient recovery, potentially due to the integration of viral RNA into the human genome [26,27,28].

No fatalities were observed among the enisamium group in the ITT population, and all patients in the enisamium group reached the primary endpoint within 21 days. By contrast, three fatalities were recorded in the placebo group, and some patients in the placebo group took longer to reach the primary endpoint. The best patient improvement was observed when enisamium was administered within 5 days (Figure 4E), which is in line with observations for other antivirals, such remdesivir [21,29], which are also most efficacious when administered early in infection, including in patients with other medical issues [30].

Our study design here was different from that of the influenza patient study [15], which was necessitated due to the lack of disease understanding at the start of the COVID-19 pandemic, the large number of trials that were conducted at the time, and additional guarantees to ensure patient health. We therefore designed our study with the inclusion of an interim analysis based on the recommendations of Bauer and Köhne [20]. In our interim analysis, no significant effect of enisamium treatment was observed among patients with an SR = 5 (hospitalized but no additional oxygen support), suggesting that the effect of enisamium may be linked to a specific group of hospitalized patients who needs non-invasive oxygen support. Due to the rapidly changing medical landscape in 2020–2021 and measures to best protect patient health, we stopped the recruitment of patients with an SR = 5 and focused on the recruitment of patients with an SR = 4. Subsequent analyses and statistical calculations were performed according to the principles of an applied adaptive research design, and an inflation of the level of significance was performed to correct for the interim analysis.

Our study was double-blinded and conducted at 14 centers, limiting bias in the observed outcomes. In addition, care was taken to confirm SARS-CoV-2 infection in hospitalized patients using RT-qPCR before randomization. This ensured that our study tested the effect of enisamium on the clinical aspects of COVID-19 and that it was not limited to deciding patient enrollment solely on clinical diagnosis. However, we did not test for the presence of other respiratory viruses and microbes and did not rule out that some patients may have had secondary infections. Secondary infections have been rare among COVID-19 patients, and we do not expect these to have impacted the described observations [31].

The median age of our ITT population was 59, which is relatively young, but a fair reflection of COVID-19 patients with moderate disease. Severe COVID-19 is typically observed in senior people and the reported age distribution is, therefore, not a limitation of our study. In addition, we observed that enisamium treatment is safe to use in COVID-19 patients with moderate disease and that an orally administered treatment in capsules of 0.5 g four times a day for 168 h is well tolerated. A limitation of our randomized, multicenter study is that it was performed in one country. Future research should aim to expand the scope to multiple countries and people of diverse backgrounds.

In summary, our data suggest that for COVID-19 patients that do not require supplementary oxygen (SR = 5), standard care is sufficient to aid recovery, and enisamium does not offer significant clinical benefits. However, standard care in combination with enisamium treatment may be more effective than standard care in combination with placebo treatment in patients with moderate COVID-19 requiring additional oxygen (SR = 4). We recommend that further research be conducted to confirm that the treatment of mild COVID-19 patients results in a clinical impact in other populations. The authorities of Ukraine have approved enisamium for clinical use in Ukraine.

## Figures and Tables

**Table 1:** The 8-point severity rating (SR) scale used for this study.

SR	Definition
1	Death
2	Hospitalized, on invasive mechanical ventilation or extracorporeal membrane oxygenation (ECMO)
3	Hospitalized, on non-invasive ventilation or high flow oxygen devices
4	Hospitalized, requiring supplemental oxygen
5	Hospitalized, not requiring supplemental oxygen—requiring ongoing medical care (COVID-19 related or otherwise)
6	Hospitalized, not requiring supplemental oxygen—no longer requires ongoing medical care
7	Not hospitalized, limitations on activities and/or requiring home oxygen
8	Not hospitalized, no limitations on activities

**Table 2:** Demographic and clinical characteristics at baseline for ITT set (a).

Characteristic	All *N* = 285	Enisamium *N* = 142	Placebo *N* = 143
Age, years—median (IQR)	59 (47–65)	59 (47–65)	59 (47.5–65)
Age category—no. (%)			
—<40 year	32 (11.2)	15 (10.6)	17 (11.9)
—40–<65 years	174 (61.1)	89 (62.7)	85 (59.4)
—≥65 years	79 (27.7)	38 (26.8)	41 (28.7)
Sex—no. (%)			
—male	134 (47.0)	64 (45.1)	70 (49.0)
—female	151 (53.0)	78 (54.9)	73 (51.0)
Race or ethnic group—no. (%) (b)			
—Caucasian (white)	284 (99.6)	141 (99.3)	143 (100.0)
—Asian	1 (0.4)	1 (0.7)	0 (0.0)
Median time (IQR) from symptom onset to randomization, days	8 (6–12)	8 (5–10)	7 (5–9)
No. of coexisting conditions—no. (%)			
None	105 (36.8)	55 (38.7)	50 (35.0)
One	104 (36.5)	52 (36.6)	52 (36.4)
Two or more	76 (26.7)	35 (24.6)	41 (28.7)
Coexisting conditions—no. (%)			
Hypertension	140 (49.1)	67 (47.2)	73 (51.5)
BMI ≥ 30 kg/m^2^	94 (33.0)	47 (33.1)	47 (32.9)
Type 2 diabetes	26 (9.1)	10 (7.0)	16 (11.2)
Severity of symptoms (d)			
Median of cough severity (IQR) (c)	2 (2–2)	2 (2–2)	2 (2–2)
Median of sore throat severity (IQR) (c)	0 (0–1)	0 (0–1)	0 (0–1)
Median of shortness in breath severity (IQR) (c)	2 (2–3)	2 (2–3)	2 (2–3)
Median of headache severity (IQR) (c)	1 (0–2)	1 (0–2)	1 (0–2)
Median of diarrhea (e) severity (IQR) (c)	0 (0–0)	0 (0–0)	0 (0–0)
Median of rhinorrhea (f) severity (IQR) (c)	0 (0–0)	0 (0–0)	0 (0–0)
Median of fatigue severity (IQR) (c)	3 (2–3)	3 (2–3)	3 (2–3)
Median of myalgia severity (IQR) (c)	2 (2–2)	1 (2–2)	2 (1–2)

(a) Percentages may not total to 100 because of rounding. (b) Race and ethnic groups were reported by the patients. (c) IQR denotes interquartile range. (d) The severity of symptoms on a verbal rating scale (VRS-4). (e) This symptom was present in 16.1% patients from the enisamium group and 16.2% from the placebo group. (f) This symptom was present in 10.6% patients from the enisamium group and 13.3% from the placebo group.

**Table 3:** Median time to reach primary endpoints for all patients and patient subgroups.

Population		Group	*n*	Median				*p*-Value (One-Sided)
				Estimate	Std. Error	95% CI		
						Lower Bound	Upper Bound	
All ITT patients (SR = 4 at baseline) with age stratification	Patients <40 years	Placebo	17	9	0.65	7.73	10.27	0.009 *
		Enisamium	15	8	0.90	6.24	9.76	
		Total	32	9	0.52	7.98	10.02	
	Patients 40–<65 years	Placebo	85	11	0.33	10.36	11.64	
		Enisamium	89	10	0.40	9.22	10.78	
		Total	174	11	0.27	10.46	11.54	
	Patients ≥65 years	Placebo	41	12	0.72	10.58	13.42	
		Enisamium	38	10	0.58	8.87	11.13	
		Total	79	11	0.65	9.73	12.27	
All patients randomized within <5 days of symptom onset		Placebo	18	13	2.11	8.87	17.13	0.005
		Enisamium	15	8	0.69	6.64	9.36	
		Total	33	10	1.10	7.85	12.15	
Patients aged ≥50 years randomized within <10 days of symptom onset		Placebo	81	12	0.52	10.98	13.02	0.002
		Enisamium	73	10	0.54	8.95	11.05	
		Total	154	11	0.30	10.41	11.59	

* Obtained using a logrank test that was stratified by age category.

**Table 4:** Results for secondary endpoints by category.

Variable	Category	Placebo		Enisamium		*p*-Value (One-Sided)
		n	%	n	%	
Patients discharged on day 8	Patient not discharged	92	73.0	81	65.3	0.119
Patients discharged on day 15	Patient not discharged	18	14.3	7	5.6	0.018
Patients discharged on day 22	Patient not discharged	8	6.3	0	0.0	0.004
Patients discharged on day 29	Patient not discharged	4	3.2	0	0.0	0.063
Deterioration by 1 SR point	Patients with deterioration of 1 point by SR scale	12	8.4	3	2.1	0.016
RT-qPCR test results on day 8	SARS-CoV-2 positive on day 8	60	51.3	52	45.6	0.233
RT-qPCR test results on day 15	SARS-CoV-2 positive on day 15	8	9.2	5	5.6	0.289
RT-qPCR test results on day 22	SARS-CoV-2 positive on day 22	2	2.4	0	0.0	0.289
RT-qPCR test results on day 29	SARS-CoV-2 positive on day 29	1	1.2	0	0.0	0.494
Mortality	Diseased patients	3	2.1	0	0.0	0.125

**Table 5:** Summary of the safety and tolerability endpoints.

Parameter	Placebo Group (*N* = 289) *n* (%)	Enisamium (*N* = 293) *n* (%)
Subjects evaluated for AR/AE analysis	289	293
Number of ARs/AEs	172	229
Patients with ARs/AEs	87 (30.1)	105 (35.8)
Number of SAEs	5 (2.9)	4 (1.7)
Patients with SAEs	3 (1.04)	4 (1.37)
Patients excluded due to ARs/AEs	15 (5.2)	15 (5.1)

*N* = the number of patients in the total analyzed; *n* = the number of patients who had events; in each line, patients were included only once.

## Data Availability

The data analyzed and presented in this study are available from P.B. and A.G. upon reasonable request, providing that the request meets local ethical and research criteria. Patient data will be anonymized, and study documents will be redacted to protect the privacy of trial participants. The study protocol is available upon request. The trial was registered with ClinicalTrials.gov under NCT04682873.

## Supplementary Materials

The following supporting information can be downloaded at: https://www.mdpi.com/article/10.3390/arm92030021/s1, Table S1: Patient inclusion criteria; Table S2: Patient exclusion criteria; Table S3: Treatment with glucocorticosteroids by study group; Table S4: Time from onset of symptoms to randomization; Table S5: Data processing summary; Table S6. Medians and means of the time to clinical improvement; Table S7: The results of applying the Log Rank test to assess the differences between age categories at the stage of "blind" data review; Table S8: Results for the secondary endpoints by study group; Table S9: Results for secondary endpoints by time to event; Table S10: Summary results for symptoms severity; Figure S1: Risk assessments are more likely to achieve clinical improvement (increase in SR scores by 2 points) for patients depending on age.

## References

[1] Gorbalenya A.E., Baker S.C., Baric R.S., de Groot R.J., Drosten C., Gulyaeva A.A., Haagmans B.L., Lauber C., Leontovich A.M., Neuman B.W. (2020). The Species Severe Acute Respiratory Syndrome-Related Coronavirus: Classifying 2019-nCoV and Naming It SARS-CoV-2. Nat. Microbiol..

[2] Zhou P., Yang X.L., Wang X.G., Hu B., Zhang L., Zhang W., Si H.R., Zhu Y., Li B., Huang C.L. (2020). A Pneumonia Outbreak Associated with a New Coronavirus of Probable Bat Origin. Nature.

[3] Velásquez P.A., Hernandez J.C., Galeano E., Hincapié-García J., Rugeles M.T., Zapata-Builes W. (2024). Effectiveness of Drug Repurposing and Natural Products Against SARS-CoV-2: A Comprehensive Review. Clin. Pharmacol..

[4] Arman B.Y., Brun J., Hill M.L., Zitzmann N., von Delft A. (2023). An Update on SARS-CoV-2 Clinical Trial Results-What We Can Learn for the Next Pandemic. Int. J. Mol. Sci..

[5] Yamato M., Kinoshita M., Miyazawa S., Seki M., Mizuno T., Sonoyama T. (2024). Ensitrelvir in Patients with SARS-CoV-2: A Retrospective Chart Review. J. Infect. Chemother..

[6] Liu W., Zhang M., Hu C., Song H., Mei Y., Liu Y., Zhang Q. (2023). Remdesivir Derivative VV116 Is a Potential Broad-Spectrum Inhibitor of Both Human and Animal Coronaviruses. Viruses.

[7] Moshawih S., Jarrar Q., Bahrin A.A., Lim A.F., Ming L., Goh H.P. (2024). Evaluating NSAIDs in SARS-CoV-2: Immunomodulatory Mechanisms and Future Therapeutic Strategies. Heliyon.

[8] Tanino Y., Nishioka K., Yamamoto C., Watanabe Y., Daidoji T., Kawamoto M., Uda S., Kirito S., Nakagawa Y., Kasamatsu Y. (2024). Emergence of SARS-CoV-2 with Dual-Drug Resistant Mutations During a Long-Term Infection in a Kidney Transplant Recipient. Infect. Drug Resist..

[9] Snijder E.J., Decroly E., Ziebuhr J. (2016). The Nonstructural Proteins Directing Coronavirus RNA Synthesis and Processing. Adv. Virus Res..

[10] Snijder E.J., Limpens R., de Wilde A.H., de Jong A.W.M., Zevenhoven-Dobbe J.C., Maier H.J., Faas F., Koster A.J., Barcena M. (2020). A Unifying Structural and Functional Model of the Coronavirus Replication Organelle: Tracking down RNA Synthesis. PLoS Biol..

[11] Posthuma C.C., Te Velthuis A.J.W., Snijder E.J. (2017). Nidovirus RNA Polymerases: Complex Enzymes Handling Exceptional RNA Genomes. Virus Res..

[12] Te Velthuis A.J. (2014). Common and Unique Features of Viral RNA-Dependent Polymerases. Cell Mol. Life Sci..

[13] Te Velthuis A.J., Arnold J.J., Cameron C.E., van den Worm S.H., Snijder E.J. (2010). The RNA Polymerase Activity of SARS-Coronavirus Nsp12 Is Primer Dependent. Nucleic Acids Res..

[14] Boltz D., Peng X., Muzzio M., Dash P., Thomas P.G., Margitich V. (2018). Activity of Enisamium, an Isonicotinic Acid Derivative, against Influenza Viruses in Differentiated Normal Human Bronchial Epithelial Cells. Antivir. Chem. Chemother..

[15] Te Velthuis A.J.W., Zubkova T.G., Shaw M., Mehle A., Boltz D., Gmeinwieser N., Stammer H., Milde J., Muller L., Margitich V. (2021). Enisamium Reduces Influenza Virus Shedding and Improves Patient Recovery by Inhibiting Viral RNA Polymerase Activity. Antimicrob. Agents Chemother..

[16] Cocking D., Cinatl J., Boltz D.A., Peng X., Johnson W., Muzzio M., Syarkevych O., Kostyuk G., Goy A., Mueller L. (2018). Antiviral Effect of a Derivative of Isonicotinic Acid Enisamium Iodide (FAV00A) against Influenza Virus. Acta Virol..

[17] Walker A.P., Fan H., Keown J.R., Margitich V., Grimes J.M., Fodor E., Te Velthuis A.J.W. (2020). Enisamium Is a Small Molecule Inhibitor of the Influenza A Virus and SARS-CoV-2 RNA Polymerases. BioRxiv.

[18] Elli S., Bojkova D., Bechtel M., Vial T., Boltz D., Muzzio M., Peng X., Sala F., Cosentino C., Goy A. (2021). Enisamium Inhibits SARS-CoV-2 RNA Synthesis. Biomedicines.

[19] Bauer P., Koenig F. (2006). The Reassessment of Trial Perspectives from Interim Data--a Critical View. Stat. Med..

[20] Bauer P., Köhne K. (1994). Evaluation of Experiments with Adaptive Interim Analyses. Biometrics.

[21] Beigel J.H., Tomashek K.M., Dodd L.E., Mehta A.K., Zingman B.S., Kalil A.C., Hohmann E., Chu H.Y., Luetkemeyer A., Kline S. (2020). Remdesivir for the Treatment of Covid-19-Final Report. N. Engl. J. Med..

[22] Dutta D., Naiyer S., Mansuri S., Soni N., Singh V., Bhat K.H., Singh N., Arora G., Mansuri M.S. (2022). COVID-19 Diagnosis: A Comprehensive Review of the RT-qPCR Method for Detection of SARS-CoV-2. Diagnostics.

[23] Broberg P. (2013). Sample Size Re-Assessment Leading to a Raised Sample Size Does Not Inflate Type I Error Rate under Mild Conditions. BMC Med. Res. Methodol..

[24] Bege M., Borbás A. (2024). The Design, Synthesis and Mechanism of Action of Paxlovid, a Protease Inhibitor Drug Combination for the Treatment of COVID-19. Pharmaceutics.

[25] Bansode S., Singh P.K., Tellis M., Chugh A., Deshmukh N., Gupta M., Verma S., Giri A., Kulkarni M., Joshi R. (2023). A Comprehensive Molecular and Clinical Investigation of Approved Anti-HCV Drugs Repurposing against SARS-CoV-2 Infection: A Glaring Gap between Benchside and Bedside Medicine. Vaccines.

[26] Zhang L., Bisht P., Flamier A., Barrasa M.I., Friesen M., Richards A., Hughes S.H., Jaenisch R. (2023). LINE1-Mediated Reverse Transcription and Genomic Integration of SARS-CoV-2 mRNA Detected in Virus-Infected but Not in Viral mRNA-Transfected Cells. Viruses.

[27] Zhang L., Richards A., Barrasa M.I., Hughes S.H., Young R.A., Jaenisch R. (2021). Reverse-Transcribed SARS-CoV-2 RNA Can Integrate into the Genome of Cultured Human Cells and Can Be Expressed in Patient-Derived Tissues. Proc. Natl. Acad. Sci. USA.

[28] Azam M., Sulistiana R., Ratnawati M., Fibriana A.I., Bahrudin U., Widyaningrum D., Aljunid S.M. (2020). Recurrent SARS-CoV-2 RNA Positivity after COVID-19: A Systematic Review and Meta-Analysis. Sci. Rep..

[29] Kalil A.C., Patterson T.F., Mehta A.K., Tomashek K.M., Wolfe C.R., Ghazaryan V., Marconi V.C., Ruiz-Palacios G.M., Hsieh L., Kline S. (2020). Baricitinib plus Remdesivir for Hospitalized Adults with Covid-19. N. Engl. J. Med..

[30] Urena Neme A.P., Tran A., Victoria Guerrero M., Roa Gomez G., Rodriguez Guerra M.A. (2024). A Successful Treatment of COVID-Induced Acute Idiopathic Pancreatitis with an RNA-Polymerase Inhibitor Agent. Cureus.

[31] Russell C.D., Fairfield C.J., Drake T.M., Turtle L., Seaton R.A., Wootton D.G., Sigfrid L., Harrison E.M., Docherty A.B., de Silva T.I. (2021). Co-Infections, Secondary Infections, and Antimicrobial Use in Patients Hospitalised with COVID-19 during the First Pandemic Wave from the ISARIC WHO CCP-UK Study: A Multicentre, Prospective Cohort Study. Lancet Microbe.

