# Supplementary material for: RNA Polymerase Inhibitor Enisamium for Treatment of Moderate COVID-19 Patients: A Randomized, Placebo-Controlled, Multicenter, Double-Blind Phase 3 Clinical Trial
Source: Adv Respir Med. 2024 May 6;92(3):202–17. doi: 10.3390/arm92030021 (PMC11130936; doi:10.3390/arm92030021)
Supplement: Supplementary file 1 [file arm-92-00021-s001.zip › arm-2935560-supplementary.pdf]

Supplemental information for:

RNA Polymerase Inhibitor Enisamium for Treatment of Moderate COVID-19 Patients: A Randomized, Placebo-Controlled, Multicenter, Double-Blind Phase 3 Clinical Trial

Table S1: Patient inclusion criteria

Number	Inclusion criterion
1	Willing and able to provide written informed consent
2	Aged ≥ 18 years
3	SARS-CoV-2 infection confirmed by PCR ≤ 4 days before randomization (test should be performed in laboratories qualified for this purpose by MOH of Ukraine; confirmation of the already established diagnosis in the central laboratory is not required)
4	Currently hospitalized due to SARS-CoV-2 infection with fever defined as body temperature ≥ 37.8 °C
5	Severity Rating Scale for Clinical Status Patient state in Covid-19: score 4 (See Table 1). Note: changed after 1st interim analysis (before also score 5 was allowed)

Table S2: Patient exclusion criteria

Number	Exclusion criterion
1	Concurrent treatment with other medicine with actual or possible direct-acting antiviral activity against SARS-CoV-2 is prohibited <24 hours prior to the start of enisamium or placebo treatment.
2	Requiring mechanical ventilation at screening or it is expected within 24 hours after inclusion.
3	Expected survival time <72 hours for any reason.
4	Positive pregnancy test.
5	Breastfeeding.
6*	Presence of renal dysfunction defined as eGFR <60 mL/min, total bilirubin ≥2.0 mg/dL, TSH outside normal range and / or ASAT / ALAT above threefold upper limit of normal range.
7	Known hypersensitivity to the trial drug, the metabolites, or formulation excipient.
8	History or presence of drug or alcohol abuse.
9	History or presence of diseases of thyroid gland.
10	Parallel participation in another clinical trial with an investigational product, participation in a clinical trial within less than 6 weeks prior to visit 1.
11	Known to be or suspected of being unable to comply with the trial protocol (e.g., no permanent address, history of drug abuse, known to be non-compliant or presenting an unstable psychiatric history).
12	Legal incapacity and / or other circumstances render the subject unable to understand the trial's nature, scope, and possible impact.
13	Subject in custody by juridical or official order.

14	Subject who has difficulties in understanding the language (Ukrainian) in which the subject information (informed consent form) is given.
15	Subjects who are members of the staff of the trial center, staff of the sponsor or the clinical research organization (CRO), the investigator him- / herself or close relatives of the investigator.

*These details became known from patient´s medical history as patient was started on treatment. It was then decided to discontinue the patient from the clinical trial and censor any data.

Table S3. Treatment with glucocorticosteroids by study group.

Characteristic	All *N*=285	Enisamium *N*=142	Placebo *N*=143
Concomitant therapy — no. (%)			
Glucocorticosteroids	200 (70.2)	97 (68.3)	103 (72.0)

Table S4. Time from onset of symptoms to randomization.

Characteristic	All *N*=285	Enisamium *N*=142	Placebo *N*=143
Median time (IQR) [a)] from symptom onset to randomization, days	8 (6 – 12)	8 (5 – 10)	7 (5 – 9)

[a)] IQR denotes interquartile range.

Table S5. Data processing summary

Age categories	Total N	*N* of Events	Censored	
			N	%
<40 years	32	29	3	9.4
40 – <65 years	174	151	23	13.2
>= 65 years	79	66	13	16.5
Total	285	246	39	13.7

Table S6 – Medians and means of the time to clinical improvement

Age categories	Mean[a]				Median			
			95% CI				95% CI	
	Estimate	Std. Error	Lower Bound	Upper Bound	Estimate	Std. Error	Lower Bound	Upper Bound
<40 years	9.24	0.44	8.37	10.11	9	0.52	7.98	10.02
40 – <65 years	11.12	0.32	10.49	11.74	11	0.27	10.46	11.54

>= 65 years	12.28	0.61	11.08	13.48	11	0.65	9.73	12.27
Total	11.22	0.27	10.69	11.74	10	0.25	9.52	10.48

[a] Estimation is limited to the largest survival time if it is censored.

Table S7. The results of applying the Log Rank test to assess the differences between age categories at the stage of "blind" data review

Test	Chi-Square	df	*p*-value
Log Rank (Mantel-Cox)	14.674	2	0.0007

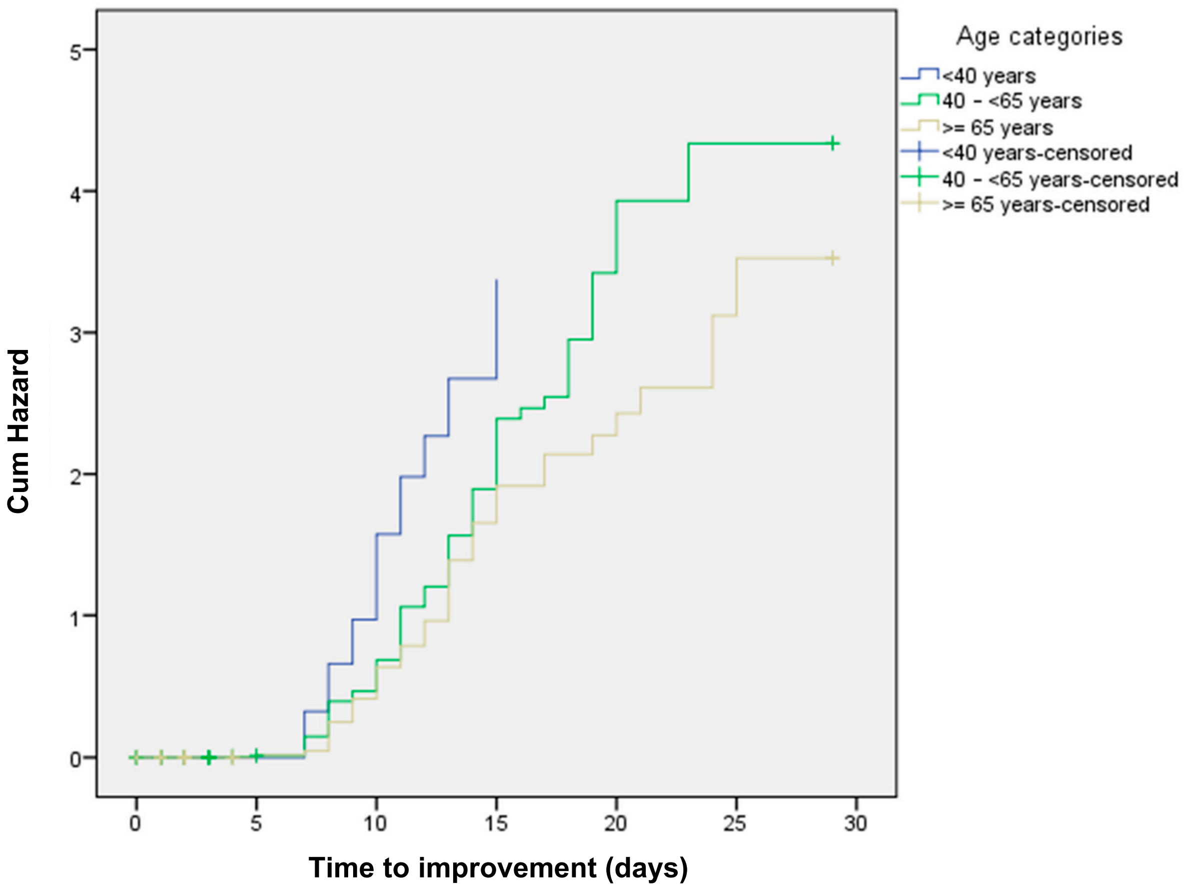

Figure S1. Risk assessments are more likely to achieve clinical improvement (increase in SR scores by 2 points) for patients depending on age.

Table S8. Results for the secondary endpoints by study group.

Variable	Group	Statistics						*P*-value (one-sided)
		n	M	Me	SD	MIN	MAX	
The sum of the scores of the subject's condition from the 2nd to the 15th day (SSR-15), points	Placebo	143	70.80	72	11.98	22	90	0.079
	Enisamium	142	72.94	75.5	11.09	47	91	
The sum of the scores of the subject's condition from the 2nd to the 29th day (SSR-29), points	Placebo	143	170.35	181	31.88	36	206	0.037
	Enisamium	142	175.85	186	26.71	112	203	
Assessment of the subject's condition on the 15th day (SR-15), points	Placebo	143	6.64	7	1.651	1	8	0.137
	Enisamium	142	6.85	8	1.48	4	8	
Assessment of the condition of the subject on the 29th day (SR-29), points	Placebo	143	7.27	8	1.589	1	8	0.109
	Enisamium	142	7.46	8	1.303	4	8	
Days alive and out of Hospital until Day 15 (DAOH-14), days	Placebo	143	2.85	3	2.551	0	9	0.112
	Enisamium	142	3.25	3	2.740	0	10	
Estimate of global efficacy by investigator, scores	Placebo	124	0.99	1	0.393	0	3	0.168
	Enisamium	123	0.93	1	0.248	0	1	
Estimate of global efficacy by subjects, scores	Placebo	124	0.90	1	0.484	0	3	0.374
	Enisamium	123	0.86	1	0.347	0	1	

Table S9. Results for secondary endpoints by time to event.

Variable	Group	*n*	Median				*P*-value (one-sided)
			Estimate	Std. Error	95% CI		
					Lower Bound	Upper Bound	
Time from first symptoms to discharge, days	Placebo	143	18	0.30	17.41	18.59	0.230
	Enisamium	142	18	0.52	16.98	19.02	
	Total	285	18	0.27	17.47	18.53	
The time from the randomization to the negative RT-qPCR test results, days	Placebo	143	10	1.50	7.07	12.93	0.190
	Enisamium	142	8	0.91	6.23	9.77	
	Total	285	10	0.84	8.35	11.65	
Time to Clinical Recovery (TTCR)*, days	Placebo	143	10	0.29	9.44	10.56	0.039
	Enisamium	142	9	0.41	8.20	9.80	
	Total	285	10	0.28	9.45	10.55	
Time to Recovery (TTR)**, days	Placebo	143	11	0.32	10.37	11.63	0.009
	Enisamium	142	10	0.34	9.33	10.67	
	Total	285	10	0.25	9.52	10.48	
Time to discontinuation of oxygen therapy, days	Placebo	143	6	0.34	5.34	6.66	0.092
	Enisamium	142	6	0.30	5.41	6.59	
	Total	285	6	0.23	5.56	6.44	

*Time to Clinical Recovery (TTCR) is the time in days from randomization (active or placebo) until normalization of fever, respiratory rate, oxygen saturation, and cough, for at least 48 hours.

**Time to Recovery (TTR) is the time in days from randomization to when the subject’s rating on the severity scale has improved from 4 to 6, 7 or 8.

Table S10. Summary results for symptoms severity*.

Variable	Category	Placebo (N = 142)		Enisamium (N = 142)		*P*-value (one-sided)
		n	%	*n*	%	
Cough severity on day 2	Reduced severity	10	7.0	12	8.5	0.412
Cough severity on day 3	Reduced severity	15	10.5	31	21.8	0.008
Cough severity on day 4	Reduced severity	30	21.0	48	33.8	0.012
Cough severity on day 5	Reduced severity	46	32.2	68	47.8	0.005
Cough severity on day 6	Reduced severity	69	48.6	76	53.5	0.238
Cough severity on day 7	Reduced severity	78	54.9	85	59.9	0.236
Cough severity on day 8	Reduced severity	83	58.5	95	66.9	0.089
Cough severity on day 9	Reduced severity	89	62.7	97	68.3	0.191
Cough severity on day 10	Reduced severity	92	64.8	99	69.7	0.224
Cough severity on day 11	Reduced severity	95	66.9	98	69.0	0.400
Cough severity on day 12	Reduced severity	96	67.6	101	71.1	0.303
Cough severity on day 13	Reduced severity	97	68.3	105	73.9	0.180
Cough severity on day 14	Reduced severity	98	69.0	108	76.1	0.116
Cough severity on day 15	Reduced severity	101	71.1	108	76.1	0.210
Shortness of breath severity on day 2	Reduced severity	14	9.9	12	8.5	0.419
Shortness of breath severity on day 3	Reduced severity	36	25.4	34	23.9	0.445
Shortness of breath severity on day 4	Reduced severity	69	48.6	62	43.7	0.238
Shortness of breath severity on day 5	Reduced severity	82	57.7	86	60.6	0.359
Shortness of breath severity on day 6	Reduced severity	95	66.9	97	68.3	0.450
Shortness of breath severity on day 7	Reduced severity	98	69.0	104	73.2	0.256
Shortness of breath severity on day 8	Reduced severity	104	73.2	113	79.6	0.132
Shortness of breath severity on day 9	Reduced severity	107	75.4	114	80.3	0.196
Shortness of breath severity on day 10	Reduced severity	111	78.2	115	81.0	0.330
Shortness of breath severity on day 11	Reduced severity	114	80.3	117	82.4	0.380
Shortness of breath severity on day 12	Reduced severity	113	79.6	121	85.2	0.138
Shortness of breath severity on day 13	Reduced severity	117	82.4	125	88.0	0.121
Shortness of breath severity on day 14	Reduced severity	117	82.4	126	88.7	0.088
Shortness of breath severity on day 15	Reduced severity	117	82.4	127	89.4	0.062
Fatigue severity on day 2	Reduced severity	17	12.0	13	9.2	0.282
Fatigue severity on day 3	Reduced severity	29	20.4	37	26.1	0.163

Variable	Category	Placebo (N = 142)		Enisamium (N = 142)		*P*-value (one-sided)
		n	%	*n*	%	
Fatigue severity on day 4	Reduced severity	51	35.9	58	40.8	0.232
Fatigue severity on day 5	Reduced severity	64	45.1	74	52.1	0.143
Fatigue severity on day 6	Reduced severity	84	59.2	85	59.9	0.500
Fatigue severity on day 7	Reduced severity	94	66.2	96	67.6	0.450
Fatigue severity on day 8	Reduced severity	101	71.1	105	73.9	0.345
Fatigue severity on day 9	Reduced severity	107	75.4	109	76.8	0.445
Fatigue severity on day 10	Reduced severity	114	80.3	109	76.8	0.282
Fatigue severity on day 11	Reduced severity	117	82.4	110	77.5	0.277
Fatigue severity on day 12	Reduced severity	117	82.4	114	80.3	0.380
Fatigue severity on day 13	Reduced severity	120	84.5	117	82.4	0.375
Fatigue severity on day 14	Reduced severity	122	85.9	119	83.8	0.370
Fatigue severity on day 15	Reduced severity	122	85.9	120	84.5	0.434
Rhinorrhea severity on day 2	Reduced severity	8	5.6	5	3.5	0.286
Rhinorrhea severity on day 3	Reduced severity	13	9.2	10	7.0	0.332
Rhinorrhea severity on day 4	Reduced severity	15	10.6	13	9.2	0.421
Rhinorrhea severity on day 5	Reduced severity	17	12.0	13	9.2	0.282
Rhinorrhea severity on day 6	Reduced severity	18	12.7	12	8.5	0.167
Rhinorrhea severity on day 7	Reduced severity	18	12.7	14	9.9	0.287
Rhinorrhea severity on day 8	Reduced severity	18	12.7	14	9.9	0.287
Rhinorrhea severity on day 9	Reduced severity	19	13.4	14	9.9	0.230
Rhinorrhea severity on day 10	Reduced severity	19	13.4	14	9.9	0.230
Rhinorrhea severity on day 11	Reduced severity	19	13.4	14	9.9	0.230
Rhinorrhea severity on day 12	Reduced severity	19	13.4	14	9.9	0.230
Rhinorrhea severity on day 13	Reduced severity	19	13.4	14	9.9	0.230
Rhinorrhea severity on day 14	Reduced severity	19	13.4	14	9.9	0.230
Rhinorrhea severity on day 15	Reduced severity	19	13.4	14	9.9	0.230
Headache severity on day 2	Reduced severity	21	14.8	31	21.8	0.083
Headache severity on day 3	Reduced severity	38	26.8	50	35.2	0.079
Headache severity on day 4	Reduced severity	57	40.1	65	45.8	0.201
Headache severity on day 5	Reduced severity	63	44.4	69	48.6	0.276
Headache severity on day 6	Reduced severity	69	48.6	72	50.7	0.406
Headache severity on day 7	Reduced severity	68	47.9	75	52.8	0.238
Headache severity on day 8	Reduced severity	75	52.8	80	56.3	0.317
Headache severity on day 9	Reduced severity	76	53.5	80	56.3	0.360
Headache severity on day 10	Reduced severity	73	51.4	83	58.5	0.142
Headache severity on day 11	Reduced severity	78	54.9	83	58.5	0.316
Headache severity on day 12	Reduced severity	78	54.9	84	59.2	0.275
Headache severity on day 13	Reduced severity	80	56.3	87	61.3	0.235
Headache severity on day 14	Reduced severity	80	56.3	89	62.7	0.167
Headache severity on day 15	Reduced severity	80	56.3	89	62.7	0.167
Sore throat severity on day 2	Reduced severity	15	10.6	12	8.5	0.343
Sore throat severity on day 3	Reduced severity	31	21.8	27	19.0	0.330
Sore throat severity on day 4	Reduced severity	42	29.6	35	24.6	0.212
Sore throat severity on day 5	Reduced severity	42	29.6	42	29.6	0.552
Sore throat severity on day 6	Reduced severity	44	31.0	45	31.7	0.500

Variable	Category	Placebo (N = 142)		Enisamium (N = 142)		*P*-value (one-sided)
		n	%	*n*	%	
Sore throat severity on day 7	Reduced severity	49	34.5	49	34.5	0.550
Sore throat severity on day 8	Reduced severity	52	36.6	49	34.5	0.402
Sore throat severity on day 9	Reduced severity	52	36.6	50	35.2	0.451
Sore throat severity on day 10	Reduced severity	54	38.0	53	37.3	0.500
Sore throat severity on day 11	Reduced severity	55	38.7	53	37.3	0.451
Sore throat severity on day 12	Reduced severity	55	38.7	55	38.7	0.548
Sore throat severity on day 13	Reduced severity	55	38.7	56	39.4	0.500
Sore throat severity on day 14	Reduced severity	55	38.7	57	40.1	0.452
Sore throat severity on day 15	Reduced severity	56	39.4	57	40.1	0.500
Diarrhea severity on day 2	Reduced severity	10	7.0	8	5.6	0.404
Diarrhea severity on day 3	Reduced severity	15	10.6	15	10.6	0.578
Diarrhea severity on day 4	Reduced severity	16	11.3	16	11.3	0.574
Diarrhea severity on day 5	Reduced severity	18	12.7	13	9.2	0.224
Diarrhea severity on day 6	Reduced severity	20	14.1	18	12.7	0.431
Diarrhea severity on day 7	Reduced severity	19	13.4	17	12.0	0.429
Diarrhea severity on day 8	Reduced severity	19	13.4	17	12.0	0.429
Diarrhea severity on day 9	Reduced severity	19	13.4	20	14.1	0.500
Diarrhea severity on day 10	Reduced severity	19	13.4	19	13.4	0.569
Diarrhea severity on day 11	Reduced severity	19	13.4	19	13.4	0.569
Diarrhea severity on day 12	Reduced severity	20	14.1	19	13.4	0.500
Diarrhea severity on day 13	Reduced severity	21	14.8	19	13.4	0.432
Diarrhea severity on day 14	Reduced severity	22	15.5	19	13.4	0.368
Diarrhea severity on day 15	Reduced severity	22	15.5	19	13.4	0.368
Myalgia severity on day 2	Reduced severity	29	20.4	25	17.6	0.325
Myalgia severity on day 3	Reduced severity	52	36.6	51	35.9	0.500
Myalgia severity on day 4	Reduced severity	80	56.3	69	48.6	0.117
Myalgia severity on day 5	Reduced severity	85	59.9	82	57.7	0.405
Myalgia severity on day 6	Reduced severity	92	64.8	85	59.9	0.231
Myalgia severity on day 7	Reduced severity	95	66.9	90	63.4	0.309
Myalgia severity on day 8	Reduced severity	96	67.6	92	64.8	0.353
Myalgia severity on day 9	Reduced severity	97	68.3	93	65.5	0.353
Myalgia severity on day 10	Reduced severity	96	67.6	94	66.2	0.450
Myalgia severity on day 11	Reduced severity	98	69.0	97	68.3	0.500
Myalgia severity on day 12	Reduced severity	97	68.3	97	68.3	0.551
Myalgia severity on day 13	Reduced severity	97	68.3	98	69.0	0.500
Myalgia severity on day 14	Reduced severity	98	69.0	98	69.0	0.551
Myalgia severity on day 15	Reduced severity	98	69.0	98	69.0	0.551

*The severity of symptoms was scored on a verbal rating scale (VRS-4): 0 scores – none / not present; 1 score – mild; 2 scores – moderate; 3 scores – severe.
